# An Appeal to Appealers

**Published:** 1895-11-02

**Authors:** 


### An Appeal to Appealers.

Now that the Hospital Sunday Fund exceeds £60,000 the managers of the medical charities in London will be in a position this year to reconsider their system of raising money. We have found, in the course of our efforts on behalf of the hospitals through the Sunday Fund, that some of the most liberal givers are getting angry with hospital secretaries, or at least with some of them. During the last ten years the supporters of hospitals have begun to take a much more intelligent interest in the work done by them. Their knowledge has increased; the means at their disposal of ascertaining the relative value, cost, and volume of the work of each institution are much greater than formerly; and contributions to hospitals are in consequence given or withheld on definite grounds. It has been the practice of some secretaries to issue appeals asking for much larger sums of money than are required, and to put into those appeals figures which are notable rather for exaggeration than precision. Those hospital managers who desire a ready and ample response to their appeal for funds will be wise to be more business-like and definite in the statements made. With the view of helping the hospitals and their supporters, we propose to deal with these appeals, or some of them, as occasion may arise, and to correct the figures where such a step seems called for. Any corrections we may find it necessary to publish will be made in a friendly spirit, but with absolute directness; no other course will meet the case.

We are in a position to know that there is abundance of money available for charities at the present time in this country, but those who are able to give most liberally have become a little shy of appeal literature as it exists at present. The writers of the appeals approach the question from a wrong point of view very often. They forget the dignity which is due to the excellence of the work they represent, and to its paramount necessity to the whole community. If one asks a man of business to join in some enterprise, the first thing the latter requires is evidence that the statements submitted to him will bear the closest investigation. Many secretaries believe, as we once believed, that it is necessary to show an enormous deficiency in order to awaken the sympathy of the person appealed to. The late Mr. Spurgeon told us years ago that the very large sums he raised were all raised because of the standpoint from which he made his appeal. He had ascertained that the creation of these great deficits was often scarcely honest, and he asserted that it was never morally defensible. Mr. Spurgeon, therefore, wrote his requests for aid from a different standpoint. He described the work and its cost, and added that when so much money was forthcoming, but not till then, so much work would be undertaken and successfully done.

Many correspondents have assured us during the last two or three years that their appeals have not brought in anything like the money they used to do. We are quite aware that this is the case, and we welcome the fact, because it proves that money is being given or withheld upon intelligent grounds. Seeing, therefore, that the old form of appeal is now, in practice, a failure, we invite the managers of every reputable charity to amend their literature and re-cast its form. Let them adopt Mr. Spurgeon's methods; let them point out the needs of the work which they wish to undertake; and let them decline to run into ruinous debt, whilst they demand as a right the subscription of the necessary money to enable the charitable enterprise for which they are responsible to flourish and extend.

A new departure in the method of appeals like that here suggested will awaken new interest and win public confidence. Therefore let it be thoroughly tried, and we have no doubt but that the result will astonish many who at the present time are disheartened by the comparative failure with which their best efforts have often been attended.

